# Molecular insights into the dynamic modulation of bacterial ClpP function and oligomerization by peptidomimetic boronate compounds

**DOI:** 10.1038/s41598-024-51787-0

**Published:** 2024-01-31

**Authors:** Bruno Alves França, Sven Falke, Holger Rohde, Christian Betzel

**Affiliations:** 1 Institute of Biochemistry and Molecular Biology, Laboratory for Structural Biology of Infection and Inflammation, University of Hamburg, c/o DESY, Build. 22a, Notkestraße 85, 22607 Hamburg, Germany; 2 Center for Free-Electron Laser Science CFEL, DESY, Notkestraße 85, 22607 Hamburg, Germany; 3 Institute of Medical Microbiology, Virology and Hygiene, University Medical Center Hamburg-Eppendorf, Martinistraße 52, 20246 Hamburg, Germany

**Keywords:** Biochemistry, Structural biology

## Abstract

Bacterial caseinolytic protease P subunit (ClpP) is important and vital for cell survival and infectivity. Recent publications describe and discuss the complex structure–function relationship of ClpP and its processive activity mediated by 14 catalytic sites. Even so, there are several aspects yet to be further elucidated, such as the paradoxical allosteric modulation of ClpP by peptidomimetic boronates. These compounds bind to all catalytic sites, and in specific conditions, they stimulate a dysregulated degradation of peptides and globular proteins, instead of inhibiting the enzymatic activity, as expected for serine proteases in general. Aiming to explore and explain this paradoxical effect, we solved and refined the crystal structure of native ClpP from *Staphylococcus epidermidis* (*Se*), an opportunistic pathogen involved in nosocomial infections, as well as ClpP in complex with ixazomib at 1.90 Å and 2.33 Å resolution, respectively. The interpretation of the crystal structures, in combination with complementary biochemical and biophysical data, shed light on how ixazomib affects the ClpP conformational state and activity. Moreover, SEC-SAXS and DLS measurements show, for the first time, that a peptidomimetic boronate compound also induces the assembly of the tetradecameric structure from isolated homomeric heptameric rings of a gram-positive organism.

## Introduction

The caseinolytic protease P (ClpP) subunit is a compartmentalized structure with two stacked heptameric rings that form a central chamber containing 14 identical catalytic sites sequestered by two protective axial pores^1^. In nature, ATPases, chaperones from the AAA+ superfamily^2^, modulate the function of ClpP^3^, controlling the pore access to hinder promiscuous proteolysis in the intracellular medium, stabilizing ClpP in its active state, and selecting the appropriate substrate to be degraded^4^.

The regulated activity of the ClpP-ATPase complex plays a key and vital role in protein turnover and homeostasis in bacteria, as this proteolytic machine degrades unfolded, misfolded, and distinct regulatory proteins^5^. In this context, the discovery and characterization of non-enzymatic activators make ClpP relevant for drug discovery investigations, aiming at the identification of novel and effective antimicrobial compounds and corresponding new therapies. The chaperone-free ClpP activation results in cellular self-digestion^6^, and this molecular mechanism has been already explored for the treatment of infections caused by multi resistant organisms, and even by dormant cells^7^. For example, the use of the natural antibiotic, acyldepsipeptide (ADEP) and its derivatives, in combination with rifampicin, a traditional ansamycin antibiotic, promoted the complete depletion of biofilms in vitro and in a mouse model^7^. ADEP, along with other ClpP modulators such as ureadepsipeptides^8^ and ZG180^9^, replace the modulation by ATPase via binding to the allosteric sites of ClpP known as “hydrophobic pockets”^10^. Those compounds also induce ClpP to be “locked” in its active state with enlarged axial pores^11^. The main advantage here, in terms of an antimicrobial effect, is that there is not any substrate selection, since the access to the protein’s catalytic chamber is independent of the regulation of a chaperone^12^. Thus, an uncontrolled protein degradation takes place inside the microbial cell, which affects cellular division, motility, and other biochemical processes essential for survival and growth^13^.

However, all data published hitherto show that the mechanism and structural aspects behind the dysregulation of ClpP activity still require further investigation. In this regard, peptidomimetic boronates, traditionally known as canonical serine protease inhibitors^14^, are worth being considered. The empty *p* orbital of boron allows it to coordinate to heteroatoms like oxygen and nitrogen, forming reversible covalent bonds with nucleophilic residues^15^ like the active serine (Ser98) of ClpP. Besides that, the peptidomimetic portion of those boronates interacts, via hydrogen bonds, with several amino acid residues in the catalytic clefts of ClpP. Those interactions are important for the stabilization of the tetradecameric active state of ClpP, as shown by previously published crystal structures in combination with isothermal titration calorimetry (ITC) measurements^16,18^.

Experiments﻿ with bortezomib, a well-known proteasome inhibitor applied as a drug for cancer treatment^17^, unveil that the activity of ClpP from *Thermus termophilus* (*Tt*) is affected by a peptidomimetic boronate in a paradoxical way^18^. The degradation of small oligopeptides increases, at low protein-to-ligand ratios, until a maximum point, and afterwards, the activity starts being reduced and inhibited as more ligand is added; proteolysis is otherwise directly proportional to the concentration of bortezomib. This kind of ambivalent activity modulation  caused by that compound is described as organism dependent: ClpP from *Escherichia coli*, for example, is only inhibited by bortezomib^18^.

In the present work, we analyzed, by selecting the ClpP from *Staphylococcus epidermidis* (*Se*ClpP), the influence of another peptidomimetic boronate, ixazomib, on protein structure and function. Our data and results obtained from X-ray crystallography, enzymatic assays, ITC, and nanoDSF rationalize how the ligand affects the substrate degradation performed by ClpP, from small oligopeptides to globular proteins. Moreover, our SEC-SAXS and DLS data reveal the impact of ixazomib in the oligomerization of *Se*ClpP.

## Results and discussion

**Figure 1:**
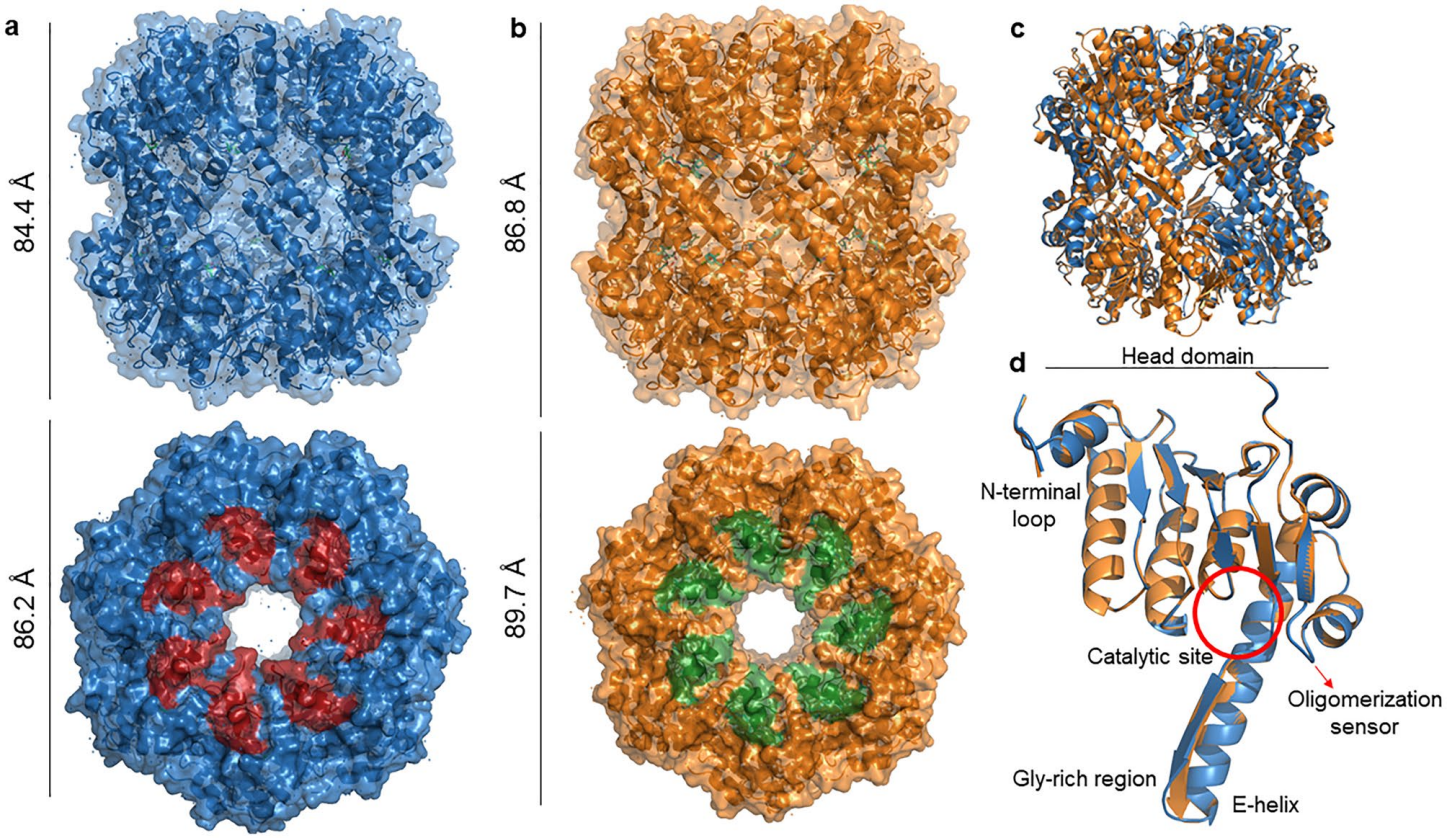
Crystal structures of ClpP from *Staphylococcus epidermidis*: apo *Se*ClpP and *Se*ClpP-ixazomib complex. The structures consist of two stacked heptameric rings that contain 14 identical catalytic sites in a central chamber. (**a**) Front and top views of apo *Se*ClpP; (**b**) front and top views of *Se*ClpP-ixazomib complex. In both cases, the protein dimensions are displayed. (**c**) The superposition of both tetradecamers demonstrates that there is not any significant three-dimensional difference between them. (**d**) Superimposition of monomers from apo *Se*ClpP (blue) and *Se*ClpP (orange) and their main parts.

The crystal structures of native *Se*ClpP (Fig. 1a) and *Se*ClpP-ixazomib (Fig. 1b) complex were refined to 1.90 Å and 2.33 Å, respectively. Further information related to X-ray data collection and refinement statistics can be found in Supplementary Table S1.

The superimposition of both tetradecameric structures (Fig. 1c) displays that the complex is slightly more extended than the apo *Se*ClpP, and the Cα RMSD is 1.63 Å for 1451 atoms. Moreover, as highlighted before, *Se*ClpP has a topology that is conserved among different source organisms: 14 monomers placed in two sevenfold symmetric rings forming, a 300kDa barrel-like structure with a central catalytic chamber protected by axial pores surrounded by allosteric sites, or “hydrophobic pockets”. These sites are depicted in red (apo *Se*ClpP) and green (*Se*ClpP-ixazomib complex) (Figs. 1a, 1b).

When *Se*ClpP monomers from both structures are compared (Fig. 1d), the Cα RMSD is 0.12 Å for 164 atoms. In both cases, the monomers are composed of a common structure consisting of the following main parts (Fig. 1d)^19^: E-helix with a Gly-rich region, catalytic site, head domain, oligomerization sensor, and N-terminal loop.

**Figure 2:**
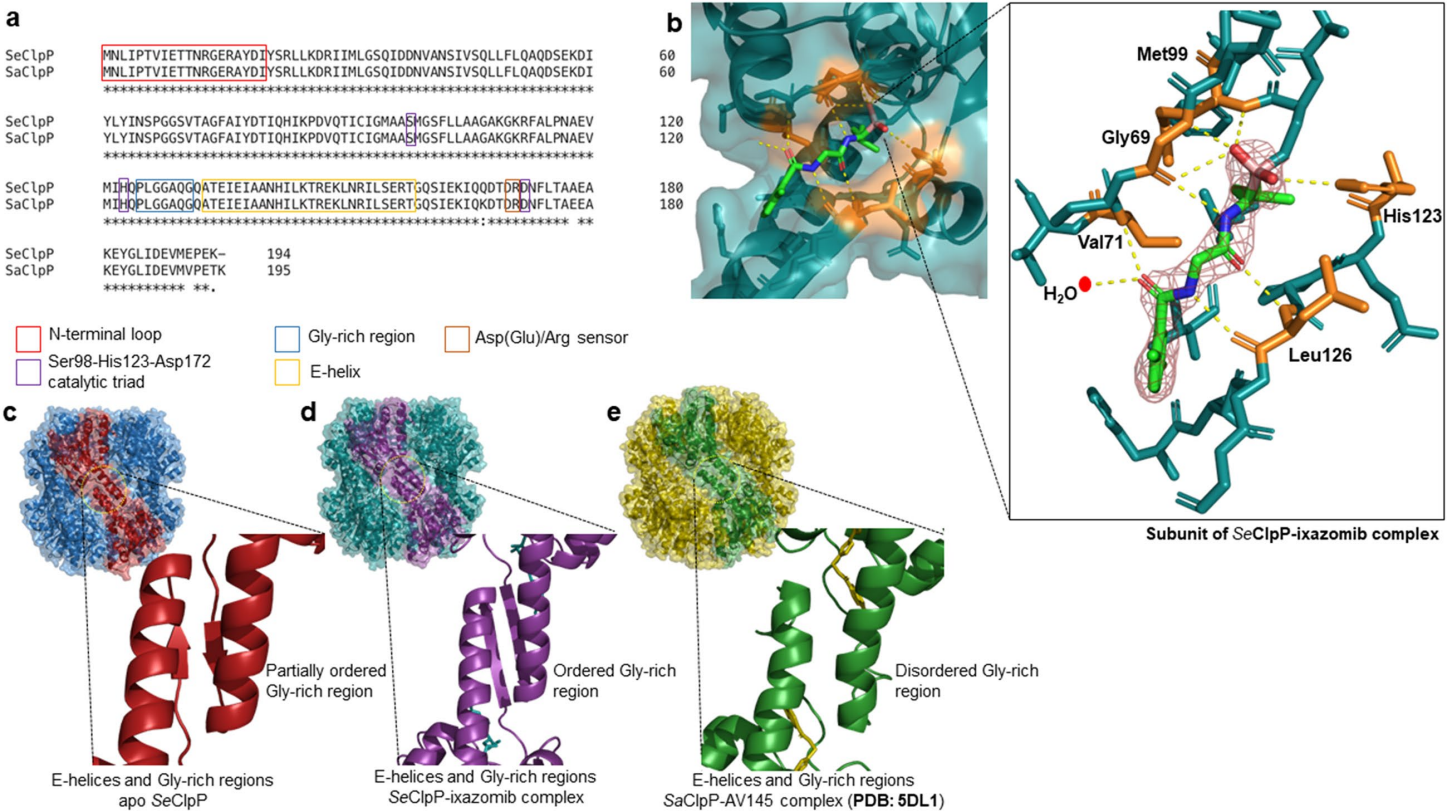
(**a**) *ClustalW* sequence alignment with *Se*ClpP and *S. aureus* ClpP (*Sa*ClpP) amino acid sequences in FASTA format; (**b**) catalytic site of a monomer from the *Se*ClpP-ixazomib complex and interactions between ixazomib and residues in the catalytic cleft. The amino acids that form hydrogen bonds with the ligand are labeled and colored in orange. Ixazomib is shown with the 2Fo-Fc electron density at 1.5σ. All the ClpP monomers bound to the ligand are shown in Supplementary Fig. S1. Comparison between the Gly-rich regions of different crystal structures of ClpP: (**c**) apo *Se*ClpP with its partially disordered Gly-rich region; (**d**) *Se*ClpP-ixazomib complex with its ordered Gly-rich region (two antiparallel beta-strands); (**e**) *Sa*ClpP-AV145 complex with its disordered, or unstructured, Gly-rich region.

Despite the similarity between the overall structures and the high homology between amino acid sequences of ClpPs from different species (Fig. 2a), a closer look into the monomers exhibits a difference in the Gly-rich region. The structure in this region seems to be more ordered when ixazomib is present. Figure 2c shows that the apo *Se*ClpP has a more disordered Gly-rich region with an unstructured loop and two small antiparallel beta-strands formed by two subunits from opposing heptameric rings. On the other hand, the boronate compound seems to induce the formation of longer antiparallel beta-strands (Fig. 2d) without a disordered loop, which results in a higher stabilization of the two stacked heptameric rings.

Other examples confirm how relevant is the flexibility of the Gly-rich region to the ClpP structure. In the compressed^20^ and compact^20^ states, both inactive states of ClpP, this region is completely unfolded, resulting in less polar interactions between the heptamers. The lack of activity in those conformations also points that the degree of order of the Gly-rich region is related to the protein's function.

Moreover, till now, some compounds are known to disrupt the tetradecamer, when covalently or non covalently bound to the catalytic sites^21^, and others that elongate or kink (Fig. 2e) the Gly-rich region but keep the heptameric rings stacked. Ixazomib, for example, is a compound that does not affect the integrity of the barrel-like structure, as also observed in ClpPs bound to peptides or other peptidomimetic compounds^16,22^.

When not bound to an ATPase or a non-enzymatic modulator, the ClpP subunit acts as a peptidase, since the closed axial pores block the access of globular proteins^23^. In any case, even the degradation of small oligopeptides depends on some structural factors, and the most important one is the orientation and positioning of the catalytic triads formed by Ser98, His123, and Asp172^24^. The protein’s mode of action corresponds to that of a chymotrypsin-like protease, and it is described in two main steps: acylation and deacylation^25^. Firstly, the nucleophilic Ser98 attacks the substrate, resulting in a tetrahedral intermediate and then a subsequent covalent acyl-enzyme, after the C-terminal fragment is released. In the last stage, a second tetrahedral intermediate is formed, and the release of the N-terminal portion proceeds, after the hydrolysis of the acyl-enzyme. For this reaction to occur, it is generally accepted that the His123 acts as a base, obtaining a proton from the Ser98 to activate it. The proper alignment of the catalytic triad is intrinsically associated with the conformation and orientation of the E-helix and the Gly-rich region. Because of the flexibility of this region^26^, a transition between the extended, compact, and compressed states is possible. The shift to one of these three states promotes a particular change in the orientation of His123. In compact and compressed forms, this amino acid residue is flipped, becoming more distant from the Ser98 (4 to 6 Å apart).

**Figure 3:**
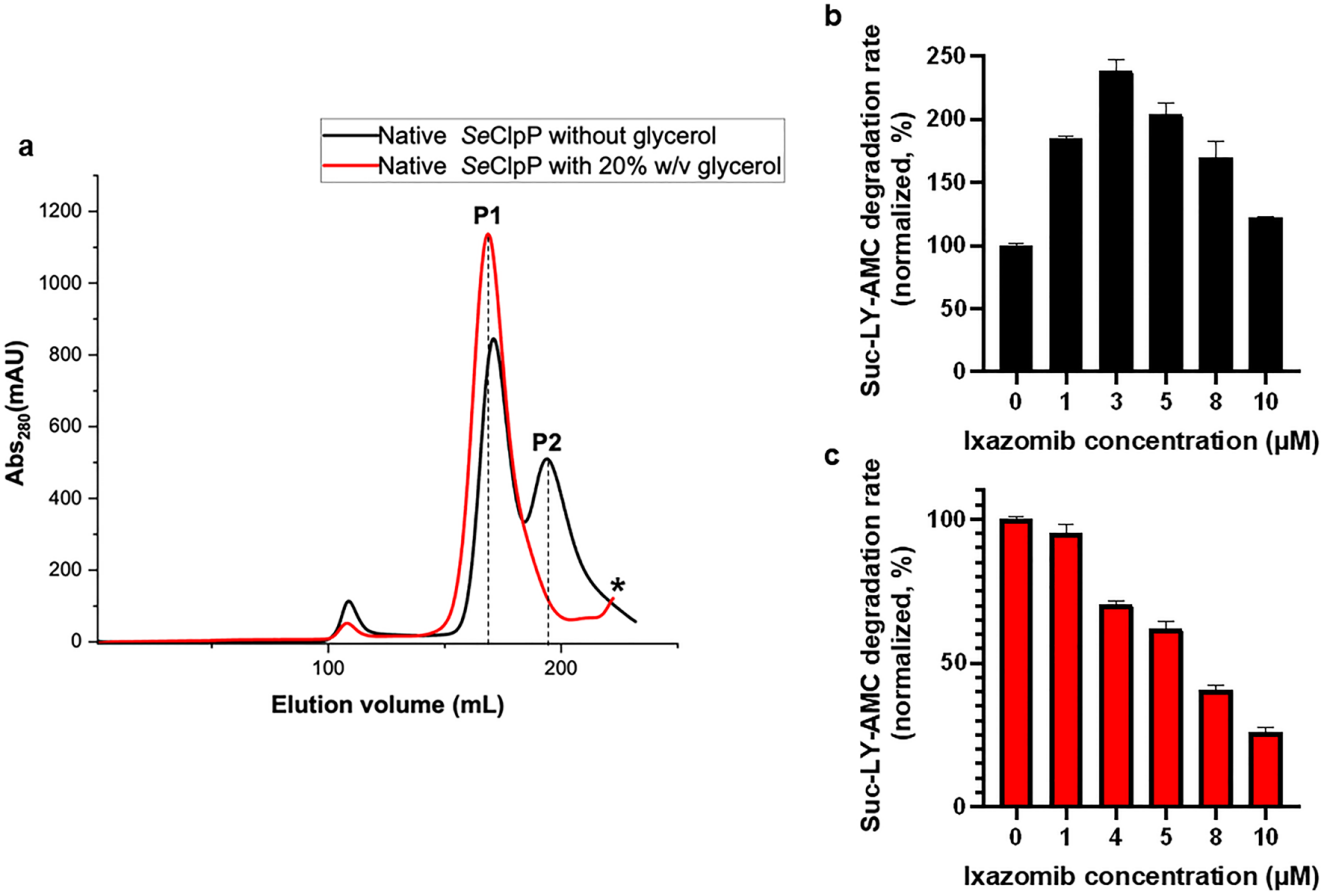
(**a**) Chromatograms after size-exclusion chromatography (SEC) with *Se*ClpP sample free from glycerol (black curve) and supplemented with 20% w/v glycerol (red curve). As seen here, the use of the triol avoids the formation of heptamers (P2). When glycerol is not added to the protein sample, both tetradecameric (P1) and heptameric (P2) species are present. Suc-LY-AMC degradation rate vs. ixazomib concentration using reaction mixtures without glycerol (black bars) ( **b**) and with 20% w/v glycerol (red bars) (**c**). All assays were performed with experimental triplicates. In Supplementary Fig. S2, curves of peptidase activity (fluorescence unit) vs. time, at different ixazomib concentrations, are presented. * Only a low amount of small oligomers of ClpP can be seen in the protein sample containing 20% w/v glycerol.

As already stated, the boron atom of ixazomib is covalently bound to Ser98, while the peptide-like portion of the ligand is stabilized by forming hydrogen bonds with amino acids in the active site of ClpP (Fig. 2b), which cause to some extent cooperative structural arrangements. This interpretation is supported by enzymatic assays we performed with the substrate Suc-LY-AMC and ixazomib at low concentrations (Fig. 3b). In a range of protein-to-ligand ratios between 1:1 to 1:3, the peptidolysis increases, and from 1:5, it starts decreasing, as the inhibitory effect is predominant.

Besides the importance of a structural alignment of the amino acids Ser98-His123-Asp172 for ClpP activity, the influence of ixazomib towards the Gly-rich regions is crucial here. From the literature, it is known that an extended beta-strand conformation of these regions supports the formation of well-defined substrate binding channels^20^. It indicates that structural changes in these channels are caused by low amounts of the ligand, which results in higher peptidolytic activity. A similar effect is seen in the study reporting data about ClpP from *Thermus thermophilus* and bortezomib^18^.

However, when the same assays are performed with a *Se*ClpP sample supplemented with 20% w/v glycerol, an increase in peptidolytic activity is not observed (Fig. 3c). This simple triol is known as chemical chaperone and protein stabilizer^26^, and if used in cell lysis and purification steps, the ClpP sample becomes homogenized with a higher concentration of extended tetradecamers, as noticeable during size-exclusion chromatography (Fig. 3a, red graph), where only one peak is observed. Conversely, when *Se*ClpP is purified in the absence of glycerol, different oligomeric states, mainly tetradecamers and heptamers, are distributed into two different peaks, P1 and P2, respectively (Fig. 3a, black graph). A lower activity is observed when the sample from peak 2 (P2) is used in enzymatic assays, once Suc-LY-AMC is poorly degraded by heptameric rings^27^.

Samples from P1 peak are constituted by tetradecameric structures capable of degrading the substrate without any additional ligand. Nevertheless, in the absence of glycerol, probably there is a mixture of active and inactive tetradecamers, and when ixazomib is used, it homogenizes the glycerol-free sample by extending all the ClpP species, causing the enhancement of peptidolysis, as explained previously^28,29^.

For further enzymatic assays, we used the tetradecameric fraction of ClpP and ixazomib, aiming to understand how the function of protein–ligand complex is modulated.

**Figure 4:**
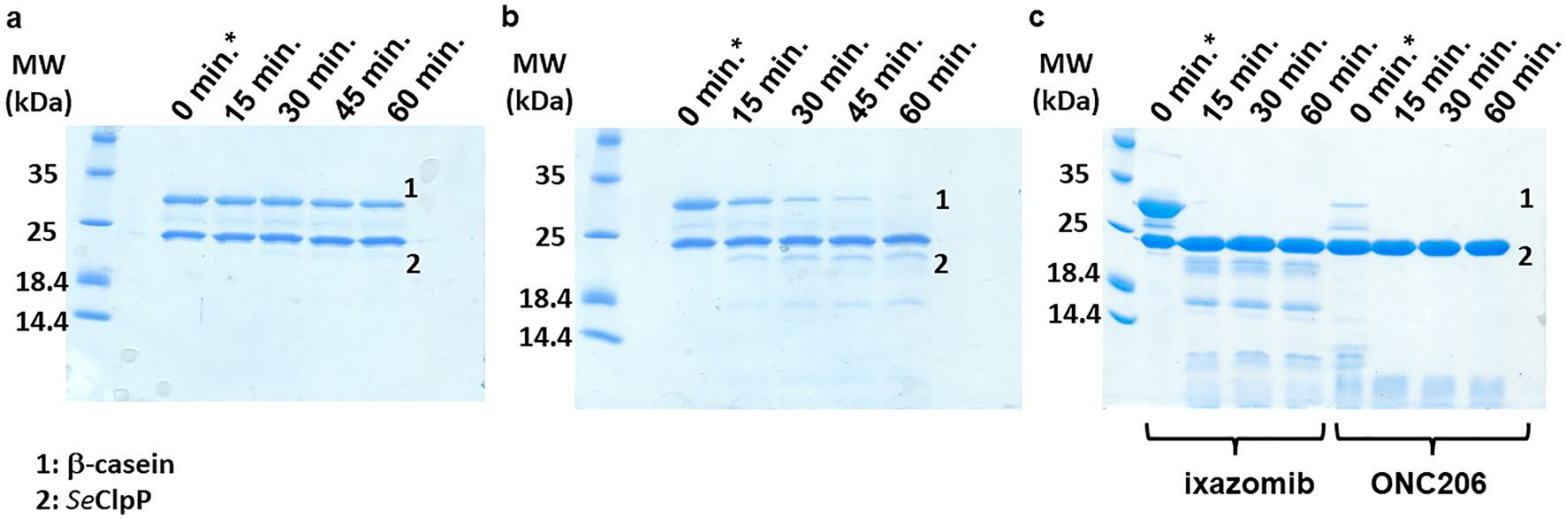
15% SDS-PAGE after reactions with β-casein monitored for 60 minutes. (**a**) ixazomib and *Se*ClpP concentrations: 200μM and 10μM, respectively; (**b**) ixazomib and *Se*ClpP concentrations: 500μM and 10μM, respectively; (**c**) Comparative experiment with ixazomib (1mM) and ONC206 (10μM), where it is noticeable that the molecular weight (MW) of product fragments vary, according to the ligand used. A possible explanation for this difference is that ixazomib occupies the catalytic sites, affecting the processive degradation of the substrate. ONC206 only binds to the allosteric regions. Degradation products of low molecular weight can be seen in (**b**) and (**c**). *t= 0 min corresponds to the time point with no incubation, but until the complete denaturation of ClpP before the SDS-PAGE, β-casein degradation happened in the quick reaction with ONC206. The entire image of each gel is found in Supplementary Fig. S3.

The relationship between the ClpP structure and function is complex. Besides the modification of the protein’s global conformation and the consequent alignment of the catalytic triads, the control of specific amino acid residues also influences proteolysis by regulating the diameter of the axial pores. In general, this structural control is facilitated by ClpP activators that bind to the “hydrophobic pockets”. Nevertheless, we confirmed that peptidomimetic boronate inhibitors also promote the dysregulation of ClpP proteolytic activity: it is directly proportional to the ixazomib concentration (Fig. 4), but it is not affected by low ligand concentrations as peptidolysis (Fig.4a). Experiments with SDS-PAGE and β-casein show that when the ligand concentration is higher than 500 µM (1:50 protein-to-ligand ratio) (Fig.4b), casein is degraded after passing through the open axial pores. In comparison to the substrate degradation in the presence of ONC206, an imipridone that modulates ClpP activation by binding to the “hydrophobic pockets”, the product profiles obtained in both cases are distinguishable (Fig. 4c). Proteolysis initiated by ONC206 results in products with lower molecular weight, as all the catalytic sites are free for the processive degradation of substrates throughout. Even being reversibly bound to ClpP, ixazomib still competes with β-casein for the catalytic triads.

The degradation of globular proteins intermediated by a serine protease inhibitor is still intriguing. So far, key amino acid residues in the hydrophobic pockets are defined as “switches” within the gating mechanism of the axial pores. To elucidate how a peptidomimetic boronate compound can support the access of bulk substrates into the active site regions, further investigations are needed. In a mutated version of ClpP from *Staphylococcus aureus* (*Sa*)^28^, the amino acid residue Tyr63, also present in *Se*ClpP, was replaced by alanine. The absence of this tyrosine results in a domino effect that shifts the orientation of Asn42 side chain, from “up” to a “down” position^28^. As a result, the electrostatic interaction between Asn42 and Tyr21 from the neighboring monomer is affected, while the N-terminal loops assume an “open” state in the pores. The same effect on Asn42 in a wild-type structure is promoted by activators that interact with Tyr63, such as ADEP. A change in the orientation of this amino acid residue also causes the same structural arrangement of Asn42^28^.

**Figure 5:**
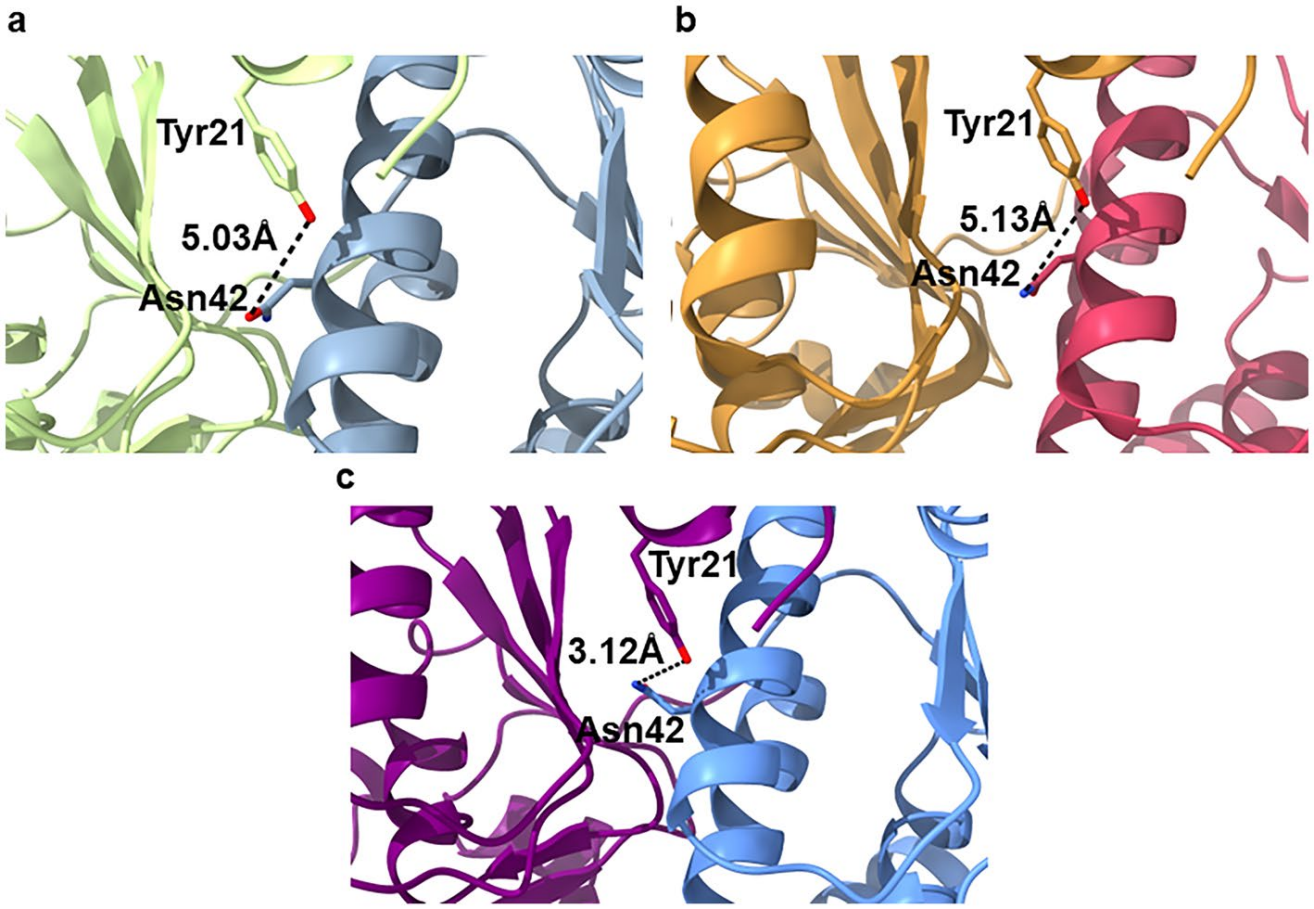
Illustration of the conformation of the Asn42 sidechain in different crystal structures of ClpP: (**a**) *Se*ClpP-ixazomib complex, (**b**) active mutant (*Sa*ClpP Y63A) (PDB ID: 5C90)^29^ of ClpP from *Staphylococcus aureus* (*Sa*), and (**c**) native *Se*ClpP. In (**a**) and (**b**), Asn42 is in a “down” position, with open pores (active for proteolysis). In contrast, in (**c**), the same amino acid residue is found in the “up” position, characteristic of closed ClpP.

A comparative analysis with the native *Se*ClpP, the *Se*ClpP-ixazomib complex, and *Sa*ClpP Y63A^29^ structures revealed that the Asn42 side chain in the complex (Fig. 5a), as well as in the mutant (Fig. 5b), is shifted to the “down” position (the hydroxyl group is approx. 5 Å distant from Tyr21). In the native protein, the residue is found in the “up” position, as expected (Fig. 5c). It indicates that the boronate compound can control the conformation of the axial pores, even if not bound to the hydrophobic pockets. Most probably, the non-covalent interactions of ixazomib play a major role in this process.

Based on the previous discussion, here we show an allosteric activation caused by the inhibitor ixazomib. This ligand, at high concentrations, controls allosterically the N-terminal loops in the pores, in a way that globular proteins can be degraded inside the inner chamber of ClpP. At the same time, at low protein-to-ligand ratios, the boronate compound induces variations in the orientation of the catalytic triad, not affecting the N-terminal loops.

**Figure 6:**
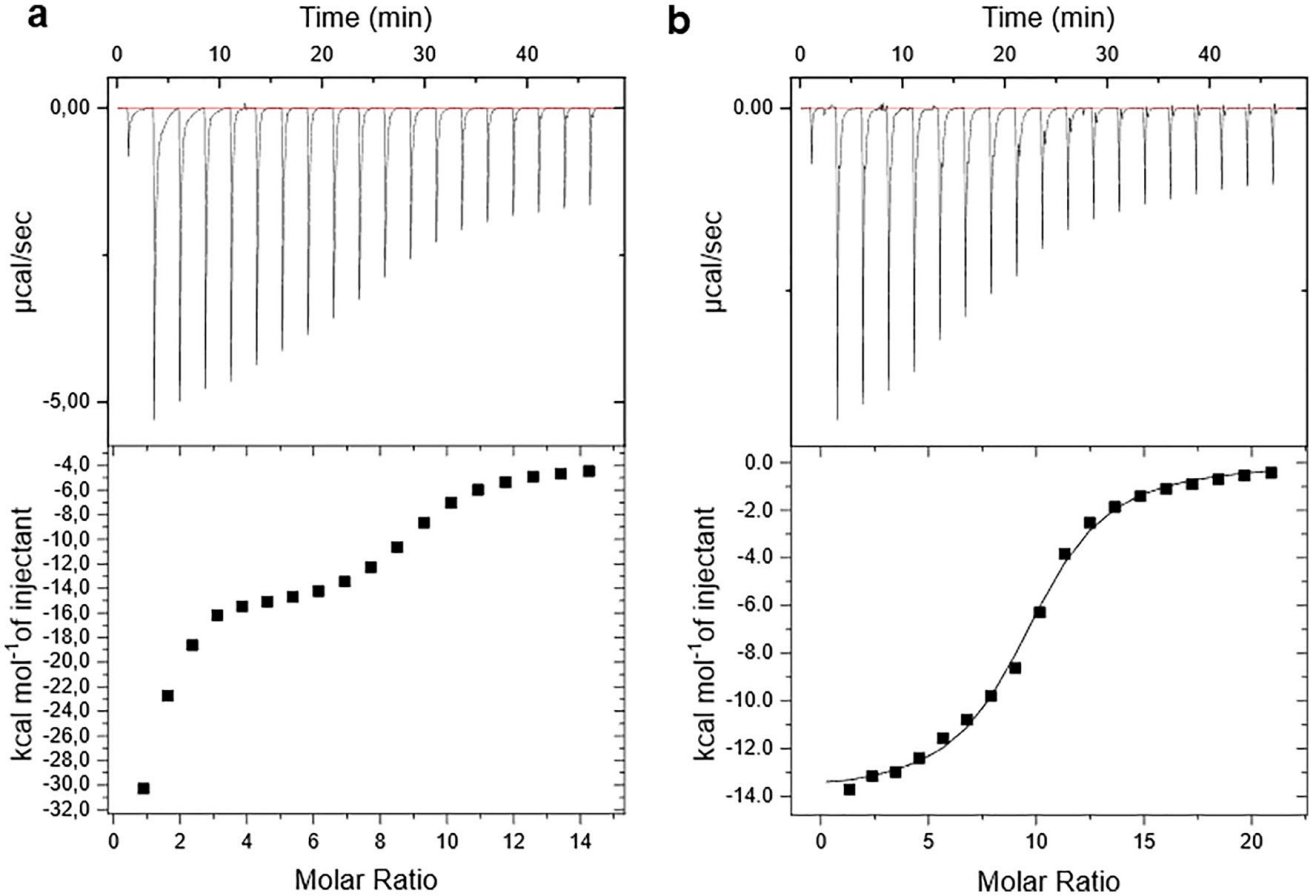
ITC measurements with *Se*ClpP and ixazomib, at different molar ratios, (**a**) in a glycerol-free environment and (**b**) with 20% w/v glycerol, both at 36°C (the same temperature set for the enzymatic assays). In the presence of glycerol, the ITC data could be fitted, and the following parameters were calculated: N = 9.53 ±0.0993 sites, K = 1.86E5 ±1.99E4 M^−1^ , $\Delta {\text{H}}$= -1.390E4 ±198.0 cal/mol, and $\Delta {\text{S}}$= -20.8 cal/mol/deg.

Additionally, our ITC analysis complements the peptidolytic assays. In the absence of glycerol (Fig. 6a), the data present a different aspect at low ixazomib concentrations, which can be a reason for the enhancement of the peptidolytic activity, as seen in Fig. 3b. This unconventional behavior was already reported in studies with ADEP^2^ where the reaction was more exothermic in the initial steps, resulted from a lower binding affinity. However, the conformational changes induced by the ligand at this stage promote positive cooperativity, increasing the binding affinity. It is important to consider how ixazomib affects the protein’s Gly-rich region and consequently, the substrate/ligand binding channels, as discussed before. Conversely, glycerol revokes the positive cooperativity, since it stabilizes the tetradecameric *Se*ClpP, also organizing the binding channels. Therefore, in this latter case, only the inhibitory effect of the peptidomimetic boronate compound is pronounced and the curve obtained from the ITC titration has a sigmoidal shape (Fig. 6b).

**Figure 7:**
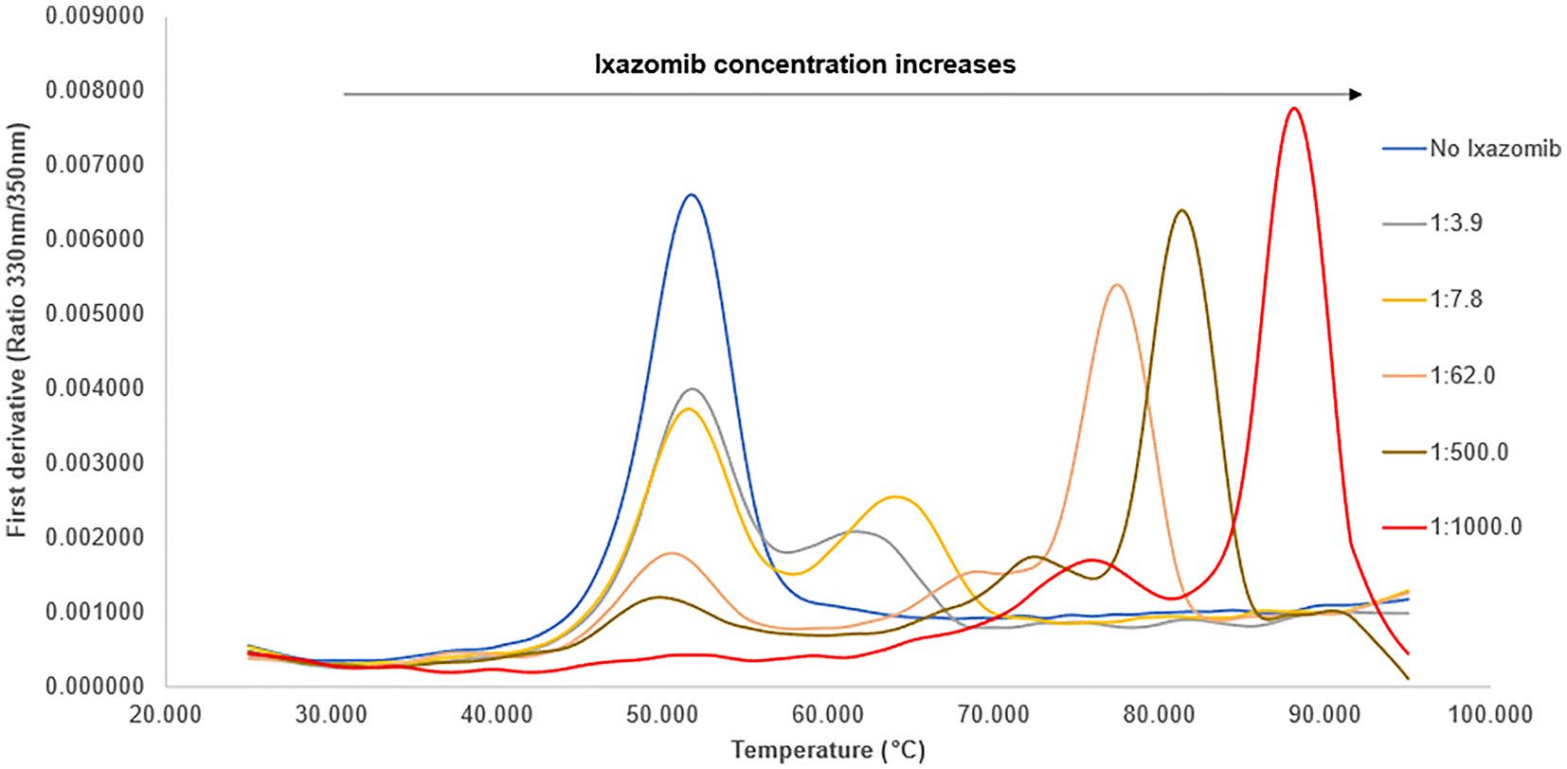
Thermal stability analysis of *Se*ClpP based on the first derivatives calculated from nanoDSF measurements. In Supplementary Fig. S4, a graph with more curves is presented, including a scan with the fluorescence intensity of samples that contain only ixazomib (at 1 mM) and buffer to discard any influence of this compound on the measurements.

nanoDSF measurements also complemented our observations. Initially, the relationship between the thermal stability of *Se*ClpP and low concentrations of ixazomib was analyzed. As shown in Fig. 7, when the protein-to-ligand ratio is 1:3.9 (gray curve), it is clear that there are two different ClpP species in solution, with melting temperatures approx. 10 units apart. In this context, the first peak, on the left, is coincident to the one of the apo *Se*ClpP (free from ixazomib). On the other hand, the second peak, on the right, is comprised by *Se*ClpP bound to the peptidomimetic boronate, and therefore more thermostable. Furthermore, we observed in this set-up that ixazomib starts to promote a conformational change that only allows the hydrolysis of relatively small oligopeptides, not globular proteins. Proteolysis is activated from higher ligand concentrations, according to the experiments in Fig. 4, which corresponds to the protein-to-ligand ratios 1:500 (brown curve) and 1:1000 (red curve). This outcome is also supported by recently published ITC measurements utilizing bortezomib as a modulator^18^, which show that the structural extension of ClpP starts at low concentrations of the ligand, but reaches a maximum point in higher amounts of the peptidomimetic boronate compound. The stabilization of the extended and more active state of *Se*ClpP results in the highest melting temperatures (approx. 80 °C and 90 °C), according to the nanoDSF measurements.

**Figure 8:**
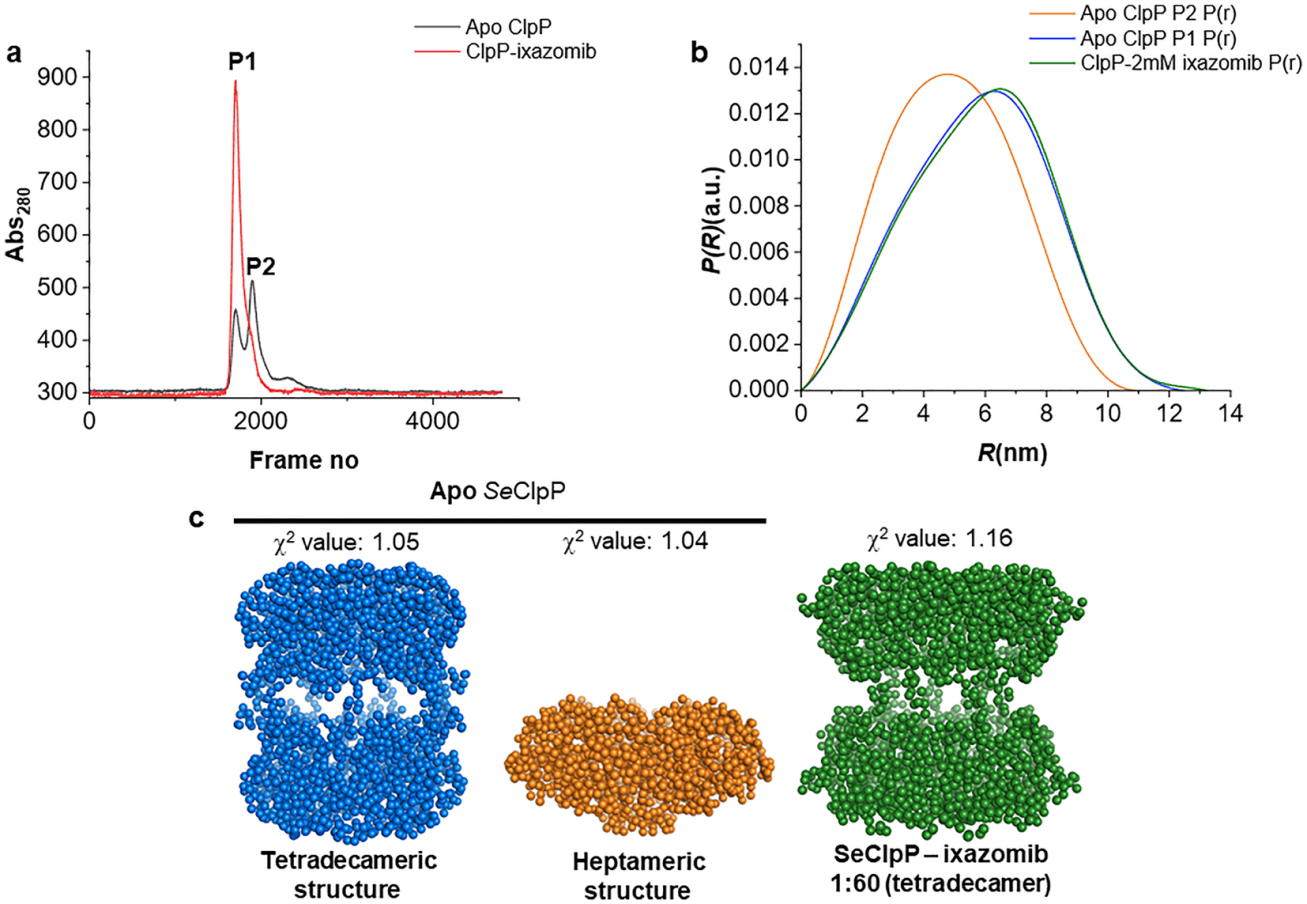
SEC-SAXS data with *Se*ClpP samples composed of heptamers and tetradecamers, as expected for protein samples without glycerol. (**a**) Chromatogram with apo *Se*ClpP (black) and *Se*ClpP sample incubated with 1 mM ixazomib (1:60 protein-to-ligand ratio; red). (**b**) Inter-atomic pair distribution functions of native *Se*ClpP P1 (blue), native *Se*ClpP P2 (orange), and *Se*ClpP-ixazomib complex (green). (**c**) Ab initio low-resolution models calculated from SAXS scattering curves of native SeClpP P1 (blue), native *Se*ClpP P2 (orange), and *Se*ClpP-ixazomib complex (green). In Supplementary Table 2, there are experimental data of SAXS measurements. Guinier plots with scattering intensity graphs (I(q) vs q) are available in Supplementary Fig. S5.

In Fig. 8, we present data from SEC-SAXS to explore the effects of ixazomib on the overall oligomerization of *Se*ClpP. Figure 8a confirms, once again, that, when glycerol is not added to the protein buffer, *Se*ClpP is found as heptamers and tetradecamers. Chromatogram represented by red color shows only one peak which contains the fractions with 300 kDa variant. It indicates and confirms that ixazomib induces the assembly of a two stacked heptameric ring structure. Previous studies with heteromeric ClpP from *Mycobacterium tuberculosis* (ClpP1P2) and peptide analogs^30^ also report a similar situation, but this protein needs a modulator to form tetradecamers anyway. In the case of *Se*ClpP, we have a homomeric protein capable of self-assembly into the active structure, even in equilibrium with another oligomeric state. Therefore, in our study, we could demonstrate this mechanism for a ClpP with identical monomers from a gram-positive bacterium.

The inter-atomic pair distribution functions in Fig. 8b demonstrate the oligomeric change induced by ixazomib as well. The heptamer species represented by the orange curve (R_G_: 3.75 ± 0.003 nm; 149.57 kDa) turn into tetradecamers (green curve; R_G_: 4.59 ± 0.002 nm, 318 kDa). In comparison to the tetradecamer isolated from the ixazomib-free solution (blue curve; R_G_: 4.47 ± 0.006 nm; 288.56 kDa), the 14-mer structure whose assembly was induced by the ligand (1:60 protein-to-ligand ratio) displays a slightly higher radius of gyration. Due to the poor solubility of ixazomib in water, the ligand amount could not be increased, as for higher concentrations of this compound, a higher DMSO concentration (a final concentration higher than 1% v/v) would be necessary, which would affect the quality of the SAXS measurements. Perhaps with increased protein-to-ligand ratios, higher R_G_ values would be observed for the *Se*ClpP-ixazomib complex, because of a more extended structure associated with a dysregulated proteolysis^2,18^.

In Fig. 8c, there are ab initio models calculated by the program GASBOR from the ATSAS suite. The models of the tetradecamer (blue) and the heptamer (orange) represent the species in the fractions P1 and P2 isolated from the sample containing native protein (black peaks) (Fig. 8a). Moreover, the green model is an illustration of the ClpP structure obtained after the P2 fraction is incubated with 2 mM ixazomib. In all cases, the χ^2^ values of approximately 1 confirm that the model fits well to the experimental data.

DLS is most useful to analyze the oligomeric shift caused by adding ixazomib, based on differences in the hydrodynamic radius (R_H_). If tetradecamers are formed from smaller oligomers, R_H_ increases.

**Figure 9:**
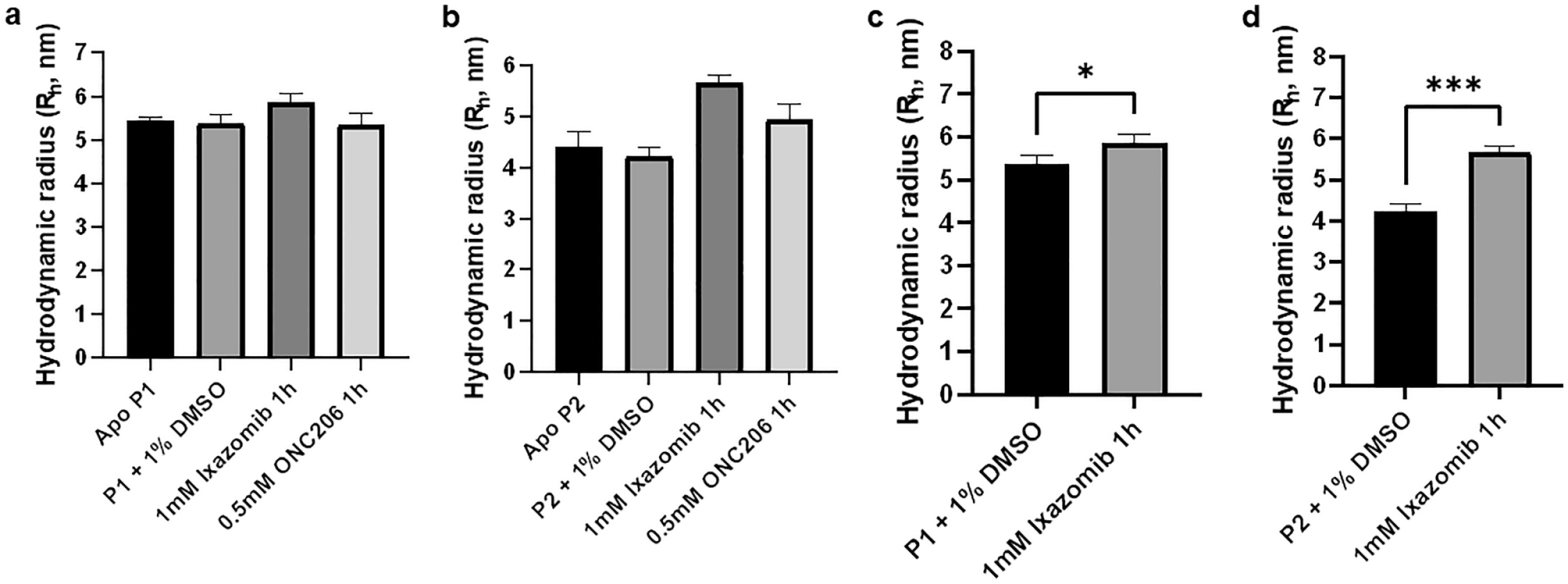
DLS measurements with apo *Se*ClpP from samples P1 (**a**) and P2 (**b**). Apo P1/P2 and P1/P2 supplemented with 1% v/v DMSO were selected as controls. 1 mM ixazomib and 0.5 mM ONC206 were incubated with the protein for 1 h, at 36 °C prior to the measurements. In (**c**,**d**), an unpaired t-test was applied to analyze how significant is the difference that ixazomib causes to R_H_ values. In both cases, the change was significant (p < 0.05): p = 0.0363 (**c**) and p = 0.0004 (**d**). The last p-value shows that a higher alteration occurs when P2 is incubated with the peptidomimetic boronate.

DLS measurements were performed, in replicates, using apo protein samples, as well as protein incubated with 1% v/v DMSO, 1 mM ixazomib, and 0.5 mM ONC206. Since this latter compound is a conventional ClpP activator, it was used in this experiment, so we could compare the outcome caused by both ligands, since ONC206 is expected to play a role in protein oligomerization. In Figs. 9a and 9b, the results corresponding to apo *Se*ClpP (P1) and apo *Se*ClpP (P2) are presented, respectively. The average R_H_ of apo *Se*ClpP P1 is approx. 5.6 nm, which is typical for tetradecamers, and the R_H_ of apo *Se*ClpP P2 is approximately 4.3 nm, confirming the presence of smaller oligomers in the sample.

The use of DMSO discarded any interference of this organic solvent in the hydrodynamic radius of *Se*ClpP in both cases. Nevertheless, ixazomib affected the protein structure in P1 and P2. In the measurements with tetradecamers (P1), the ligand promoted the extension of *Se*ClpP to approx. R_H_ = 6.0 nm (Fig. 9c). For the P2 sample, the structural change was more drastic, as R_H_ varied from 4.3 to 5.7 nm (Fig. 9d), a value slightly lower than the one observed for P1. This is additional evidence that the assembly of tetradecamers is induced by ixazomib. ONC206 was also capable of assembling larger oligomers of *Se*ClpP (Fig. 9b), but the final structures do not have a R_H_ as high as the one observed for *Se*ClpP-ixazomib complex. As that activator binds to the “hydrophobic pockets”, maybe it is more efficient to establish interactions between adjacent subunits in the same ring, resulting in independent heptameric rings.

## Conclusion

The data of the present work provide new insights about the modulation of ClpP function, dynamics, and oligomerization under the influence of boron-based peptidomimetic compounds. The high-resolution crystal structures of native ClpP and the ClpP-ixazomib complex demonstrate how the inhibitor is bound to the catalytic sites, allowing us to hypothesize the way the ligand dysregulates the proteolytic activity. Biochemical and biophysical experiments also revealed how ixazomib modulates ClpP activity and initiates global conformational alterations, particularly by affecting the Gly-rich region and the orientation of Asn42 between two adjacent ClpP monomers. An important consideration is that these alterations not only depend on the source organism of ClpP. Experimental aspects like the conformational state that is isolated after the purification steps play a major role in how ligand binding affects peptidolysis.

SEC-SAXS and DLS results showed and confirmed that the boronate inhibitor ixazomib induces the assembly of the tetradecameric structure as well. These novel results and corresponding conclusions highlight the complexity of the modulation of ClpP, showing that conformational changes in the catalytic sites also cause cooperative structural changes in more distant regions, such as the axial pores. Structural studies of this area are to some extent compromised, since the relative low electron density in this region did not allow us to resolve the flexible N-terminal loops completely. However, the opening of the gating mechanism can be inferred from the assays with β-casein, since it is the only way for a globular protein to have access to the ClpP’s catalytic chamber.

Another relevant point is how ixazomib differently affects peptidolysis and proteolysis. The explanation for the enhancement of Suc-LY-AMC degradation seems to be more straightforward, because of the structural modifications in the Gly-rich region and the cooperative effect indicated by ITC measurements at low concentrations of the ligand.

## Methods

### Heterologous expression of *Se*ClpP

ClpP gene from *Staphylococcus epidermidis* was obtained from UniProt data bank (UniProtKB: Q8CTE0) and the corresponding open reading frame (ORF) was inserted into the vector pET28a (+) (Invitrogen, USA), after codon optimization, for posterior heterologous expression with a C-terminal His-Tag in *Escherichia coli*. The resulting plasmid (Biocat GmbH, Germany) was transformed into competent *Escherichia coli* BL21(DE3) Star cells (Invitrogen, USA), and for protein expression, bacteria were grown in LB medium supplemented with 50 µg/mL kanamycin and 0.5 mM IPTG, at 20 °C for 16 h, in an orbital shaker for constant stirring at 180 rpm.

### Cell lysis and protein purification

Pellets were homogenized with buffer (A): 50 mM Tris base (pH 8.0), 100 mM NaCl, 20% w/v glycerol, or buffer (B): 50 mM Tris base (pH 8.0), 100 mM NaCl, 2 mM DTT. After sonication on ice and centrifugation, the supernatant was mixed with Ni–NTA resin (MACHEREY–NAGEL GmbH & Co. KG, Germany), previously equilibrated with (A) or (B). Protein sample eluted at 300 mM imidazole was dialyzed overnight in (A) or (B). Subsequently, *Se*ClpP sample was submitted to size-exclusion chromatography in a HiLoad 26/600 Superdex 200 preparation grade (pg) column connected to an ÄKTA Purifier 10 system (General Electric, Sweden). The inactive mutant of *Se*ClpP (S98A) was also expressed and purified as described in “Heterologous expression of *Se*ClpP” and “Cell lysis and protein purification” sections. *Se*ClpP S98A was applied to an enzymatic assay to confirm the absence of endogenous ClpP from *Escherichia coli*, as shown in Supplementary Fig. S6.

### Protein crystallization, data collection, and refinement

Needle-like crystals were obtained by applying the hanging-drop crystallization technique, using MPD as precipitant agent. The mother liquor contained 0.1 mM sodium acetate (pH 4.5), 40% v/v MPD and 40 mM calcium chloride. Crystals with dimensions of 60 µm were obtained after 20 min, and 250 µm-crystals were observed after 48 h. To analyse the structure of the *Se*ClpP-ixazomib complex, crystals were soaked with 10 mM ixazomib for 1 h before subsequent flash-freezing in liquid nitrogen. Diffraction data were collected at the beamline P11/PETRA III (DESY, Hamburg, Germany), at cryo conditions, up to 1.9 Å for apo *Se*ClpP and to 2.33 Å for *Se*ClpP in complex with ixazomib. Data merging and indexing were performed applying the XDS program package^31^ Data collection, processing and refinement parameters are summarized in Table S1 (supplementary material). The phase problem was solved by molecular replacement, using as search model the crystal structure of ClpP from *Staphylococcus aureus* (PDB: 3V5E; homology: 98.45%), and by applying the *PHASER* package from the Phenix suite version 1.20.1-4487^32^. Refinement of the apo protein structure and the ClpP-ixazomib complex was performed by using *phenix.refine*^33^. Manual model building and evaluation of refined structures were done applying the program *Coot*^34^. Stereochemical parameters were analysed via the MolProbity server^35^.

### Fluorescence assays and peptidolytic activity of *Se*ClpP

The effect of ixazomib at different concentrations on the peptidolytic activity of *Se*ClpP was analyzed via peptidolytic assays by applying the TECAN Infinite Pro 200 plate reader (TECAN, Austria) using 1 µM of protein and 100 µM of the substrate Suc-LY-AMC (Cayman Chemical, USA) in a reaction buffer with 50 mM Tris base (pH 7.5), 100 mM KCl and 2 mM DTT (C) or 50 mM Tris base (pH 7.5), 100 mM NaCl and 20% w/v glycerol (D), respectively. The effect of ixazomib on the peptidolytic activity, at different ligand concentrations, was analyzed. The ixazomib stock solution (100 mM in 100% v/v DMSO) was diluted in a way that the final DMSO concentration was kept constant to be 1% v/v.

### SDS-PAGE experiments and proteolytic Activity of *Se*ClpP

The globular protein β-casein from cow’s milk (Sigma-Aldrich, USA) was used as substrate to analyze the proteolytic activity dysregulated by ixazomib. 10 µM of ClpP were incubated with 200, 500, and 1000 µM of ixazomib, at 36 °C, under stirring at 900 rpm, in a ThermoMixer device (Thermo Fisher Scientific, USA). For each ixazomib concentration, five different tubes were prepared based on the reaction time scheme: 0 min, 15 min, 30 min, 45 min, 60 min. 7.5µL from the 0 min. sample were immediately mixed with 10 µL SDS-PAGE sample buffer and 2.5 µL DTT, being subsequently heated up at 95 °C for 10 min. The same procedure was performed with the remaining five tubes at the end of each corresponding reaction time interval. Following the step of protein denaturation, 15 µL of each sample were loaded into a polyacrylamide gel for subsequent SDS-PAGE analysis.

### NanoDSF experiments

The influence of ixazomib on the thermostability of *Se*ClpP was evaluated by applying nanoDSF. Samples with 10 µM *Se*ClpP were prepared, in replicates, with different concentrations of ixazomib in buffer C, and at least 12 µL of each one was transferred to clear capillaries (NanoTemper, Germany). The measurements were done in a temperature range between 25°C and 90°C. The raw data were automatically processed using the Prometheus NT.48 software (NanoTemper, Germany) and the resulting data were further analyzed using the MoltenProt server^36^.

### Isothermal titration calorimetry (ITC)

The interaction between *Se*ClpP and ixazomib was also assessed by applying ITC using an Auto-iTC200 microcalorimeter (MicroCal-Malvern Panalytical, Malvern, UK). Calorimetric titrations were performed using a 800 µM ixazomib solution and a 10 µM *Se*ClpP solution (buffer containing 50 mM Tris (pH 7.5), 150 mM KCl and 2 mM DTT). The heat evolved after injection of ixazomib was calculated from the integral of the measured calorimetric signal. Experiments were performed in replicates, and data were analyzed using Origin 7.0 (OriginLab, USA).

### Size-exclusion chromatography and small-angle X-ray scattering (SEC-SAXS)

*Se*ClpP incubated with ixazomib was utilized for SEC-SAXS measurements at the beamline P12 (EMBL/PETRAIII/DESY, Hamburg, Germany). Samples were prepared in a buffer containing 50 mM Tris base (pH 7.5), 150 mM KCl and 2mM DTT. For the size-exclusion chromatography, a Superdex 200 Increase 10/300 GL (GE Healthcare) column was used, and for all experiments, the concentration of the injected protein was 10 mg/mL. Eluted protein was automatically subjected to X-ray scattering data collection by utilizing a Dectris 2D photon-counting detector (PILATUS-6M) with 3.1 m sample-to-detector distance. Furthermore, SAXS data were collected at a constant temperature of 10 °C. The obtained scattering frames were visualized and processed using the ATSAS software suite^37^ and BioXTAS RAW^38^. Ab initio model of *Se*ClpP as tetradecamers (P72) and heptamers (P7) were built by using the software GASBOR^39^.

### DLS measurements

Protein samples (2.5 mg/mL) in 50 mM Tris base (pH 7.5), 150 mM KCl and 2 mM DTT were loaded into disposable plastic cuvettes with 1 mM ixazomib, 0.5 mM ONC206 or 1% v/v DMSO (as control). DLS experiments were performed applying a Wyatt Möbius laser photometer (Wyatt Technologies, USA) (532 nm and detector angle at 163.5°) at 25 °C. For each sample, 20 measurements in three independent replicates were performed, which consisted of 20 acquisitions each with an acquisition time of 20 s. Data were analysed using the software Dynamics version 7 (Wyatt Technology, USA). To evaluate how significantly ixazomib affects R_H_, *p*-values from unpaired t-tests were calculated by applying the program GraphPad Prism version 9 (GraphPad Software, USA), using an independent two-sample *t*-test (*N* = 20).

### Supplementary Information

Supplementary Information.

## Data Availability

Crystal structures of native *Se*ClpP and *Se*ClpP-ixazomib complex can be found on Protein Data Bank (PDB) under the following IDs: 8CJ4 and 8QYF, respectively. The authors confirm that further data supporting the findings of this study can be found within the article and its supplementary materials. In any case, other information that support our work are available from the main author, B. A. F., upon reasonable request. Please address eventual questions to bruno.franca@chemie.uni-hamburg.de.

## Abbreviations

RMSD: Root-mean-square deviation
DTT: Dithiothreitol
MPD: 2-Methyl-2,4-pentanediol
Suc-LY-AMC: Succinylated-leucine-tyrosine-7-amino-4-methylcoumarin
SEC-SAXS: Size-exclusion chromatography with small-angle X-ray scattering
DLS: Dynamic light scattering
