# Supplementary material for: Molecular insights into the dynamic modulation of bacterial ClpP function and oligomerization by peptidomimetic boronate compounds
Source: Sci Rep. 2024 Jan 31;14:2572. doi: 10.1038/s41598-024-51787-0 (PMC10830462; doi:10.1038/s41598-024-51787-0)
Supplement: Supplementary file 1 — Supplementary Information. [file 41598_2024_51787_MOESM1_ESM.pdf]

Molecular insights into the dynamic modulation of bacterial ClpP function and oligomerization by peptidomimetic boronate compounds

Bruno Alves França[1], Sven Falke[2] Holger Rohde[3] & Christian Betzel[1*]

[1] Institute of Biochemistry and Molecular Biology, Laboratory for Structural Biology of Infection and Inflammation, University of Hamburg, c/o DESY, Build. 22a, Notkestraße 85, 22607 Hamburg, Germany.

[2] Center for Free-Electron Laser Science CFEL, DESY, Notkestraße 85, 22607 Hamburg, Germany.

[3] University Medical Center Hamburg-Eppendorf, Institute of Medical Microbiology, Virology and Hygiene, Martinistraße 52, 20246 Hamburg.

*Corresponding author

christian.betzel@uni-hamburg.de

Supplementary information

Table S1: X-ray data collection and refinement statistics.

	***Se*ClpP-Ixazomib**	**native *Se*ClpP**
**Data collection**		
**X-ray source**	P11, PETRA III, DESY	P11, PETRA III, DESY
**Detector**	Pilatus 6M	Pilatus 6M
**Space group**	$P\ 2_1$	$P\ 2_1$
**Cell dimensions**		
***a*, *b*, *c* (Å)**	94.96, 123.16, 126.24	95.42, 123.66, 126.79
**Wavelength (Å)**	1.03	1.03
**Resolution (Å)**	47.94 – 2.33 (2.41 – 2.33)	48.21 – 1.90 (1.94 – 1.90)
**Total reflections**	856843 (85183)	1613744 (87103)
**Total unique reflections**	123893 (12335)	456211 (25528)
**Redundancy**	6.9 (6.9)	3.5 (3.4)
**Wilson B-factor (Å^2)**	40.15	27.83
**R_{meas}**	0.14 (1.30)	0.17 (1.01)
**$CC_{1/2}$**	1.00 (0.61)	0.98 (0.68)
**I/σI**	12.85 (1.56)	4.72 (0.58)
**Completeness (%)**	99.93 (99.96)	98.59 (96.16)
**Refinement**		
**Reflections used**	123871 (12336)	228269 (13047)
**Reflections used for R_{free}**	1768 (176)	2396 (137)
**R_{work}**	0.21 (0.30)	0.18 (0.25)
**R_{free}**	0.22 (0.33)	0.22 (0.29)
**No. of atoms**	19906	21199
**Protein**	19005	19446
**Ligand**	322	112
**Water**	579	1641
**Average B-factor (Å^2)**	45.98	31.38
**macromolecules**	45.75	30.88
**Ligands**	57.45	34.04
**Water**	47.13	37.09
**R.m.s deviations**		
**bond lengths (Å)**	0.01	0.008
**bond angles (°)**	1.14	0.96
**Ramachandran**		
**favored (%)**	98.25	98.46
**allowed (%)**	1.75	1.54
**outliers (%)**	0.00	0.00
**PDB ID**	8QYF	8CJ4

Statistics for the last shell are shown in parenthesis.

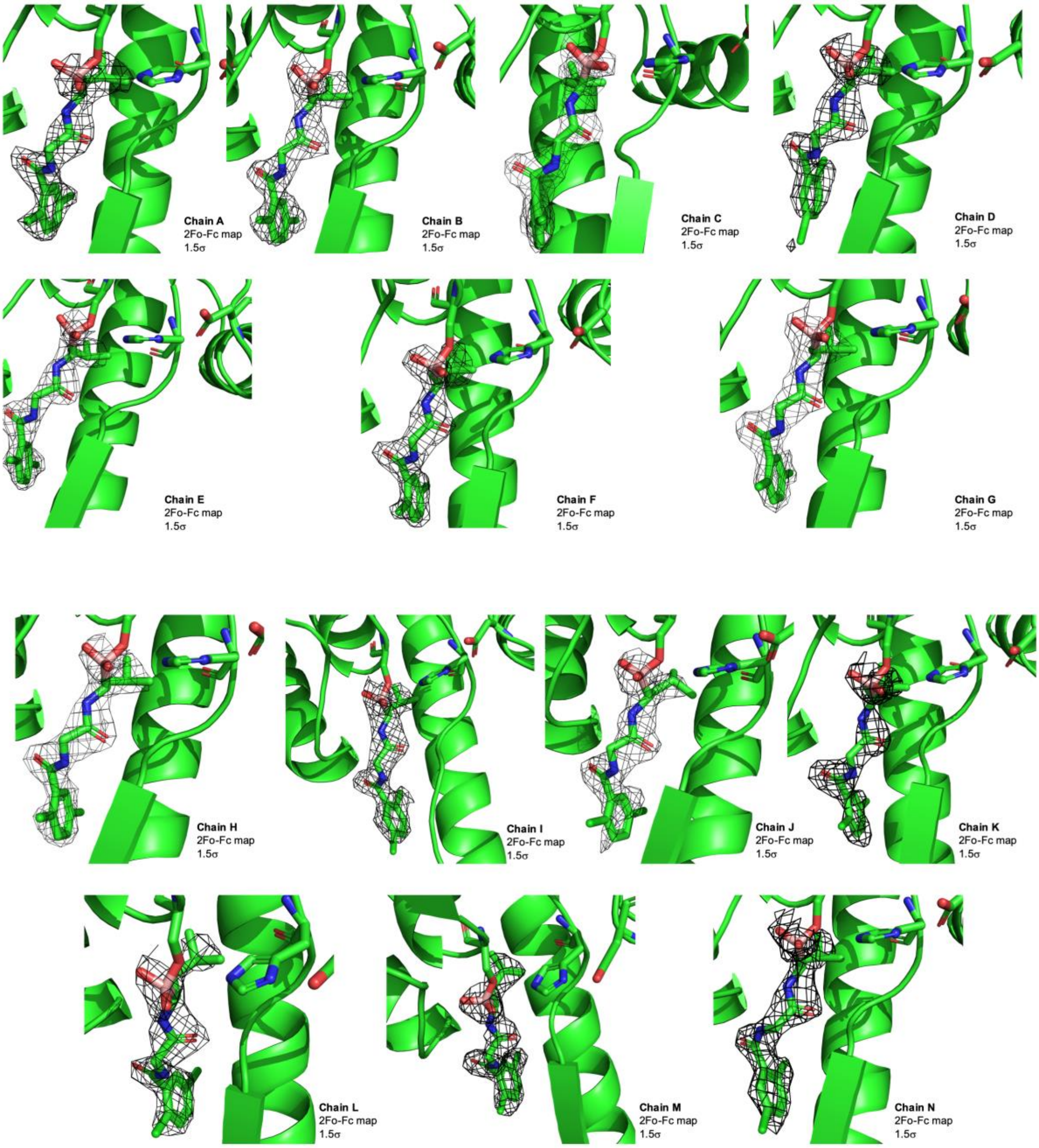

Fig. S1 – Catalytic sites of all 14 *Se*ClpP monomers in complex with ixazomib. The ligand molecule is shown with the corresponding 2Fo-Fc electron density at 1.5σ. The refined ligand occupancy varies from 0.80 to 1.0.

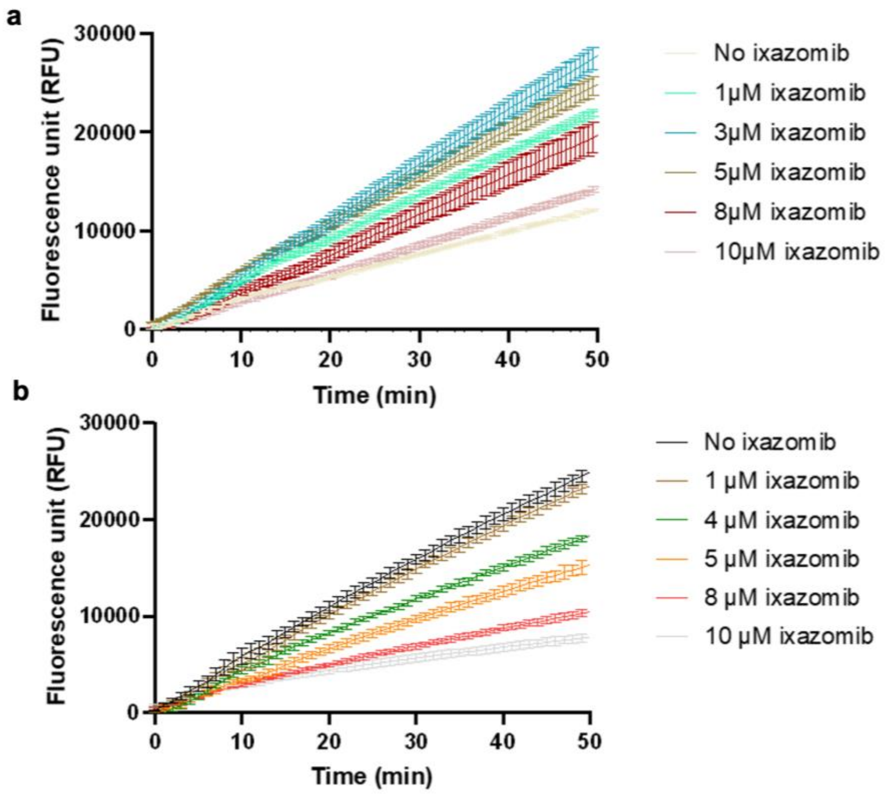

Fig. S2 – Hydrolysis of the fluorogenic peptide substrate Suc-LY-AMC (100μM) by *Se*ClpP (1μM) in the absence and presence of glycerol, (**a**) and (**b**), respectively. The influence of 20% w/v glycerol in the sample and reaction buffers is also analyzed in (**b**). In (**a**), it is noticeable that, at low concentrations of ixazomib (from 1 to 3μM), the peptidolytic activity is enhanced. For concentrations higher than 3μM, the inhibition of peptidolysis starts to occur. In (**b**), in the presence of glycerol, only protein inhibition is observed. The cleavage of Suc-LY-AMC was monitored by fluorescence (excitation = 340 nm; emission = 460 nm). Error bars represent the standard deviation of three independent experiments.

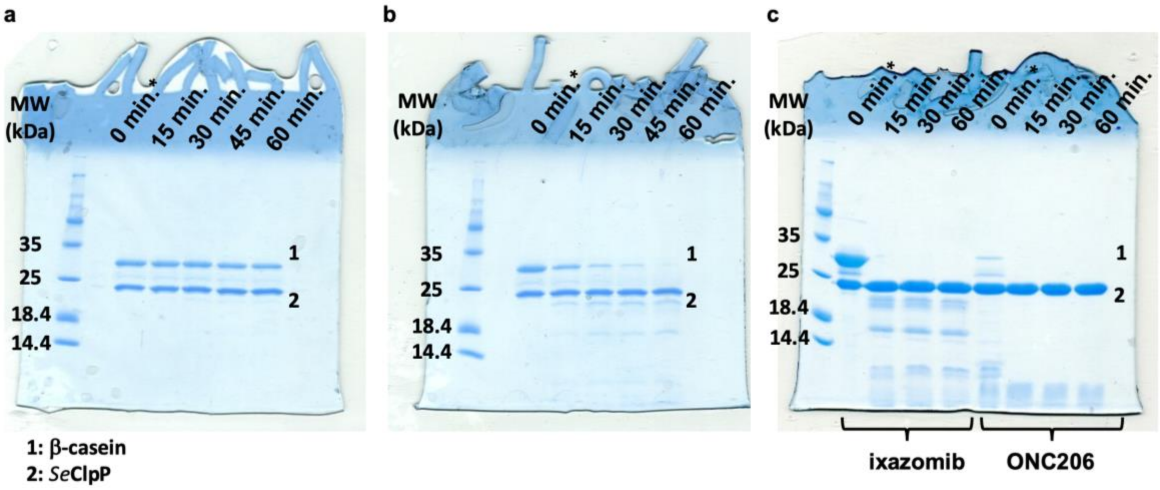

Fig. S3 - 15% SDS-PAGE (the gel limits are visible here) after reactions with β-casein monitored for 60 minutes. (**a**) ixazomib and *Se*ClpP concentrations: 200μM and 10μM, respectively; (**b**) ixazomib and *Se*ClpP concentrations: 500μM and 10μM, respectively; (**c**) Comparative experiment with ixazomib (1mM) and ONC206 (10μM), where it is noticeable that the MWs of product fragments vary, in accordance with the ligand used. A possible explanation for this difference is that ixazomib occupies the catalytic sites, affecting the processive degradation of the substrate. ONC206 only binds to the allosteric regions. Degradation products of low molecular weight can be seen in (**b**) and (**c**). *t= 0 min corresponds to the time point with no incubation, but until the complete protein denaturation prior to the SDS-PAGE, β-casein degradation happened in the quick reaction with ONC206.

a

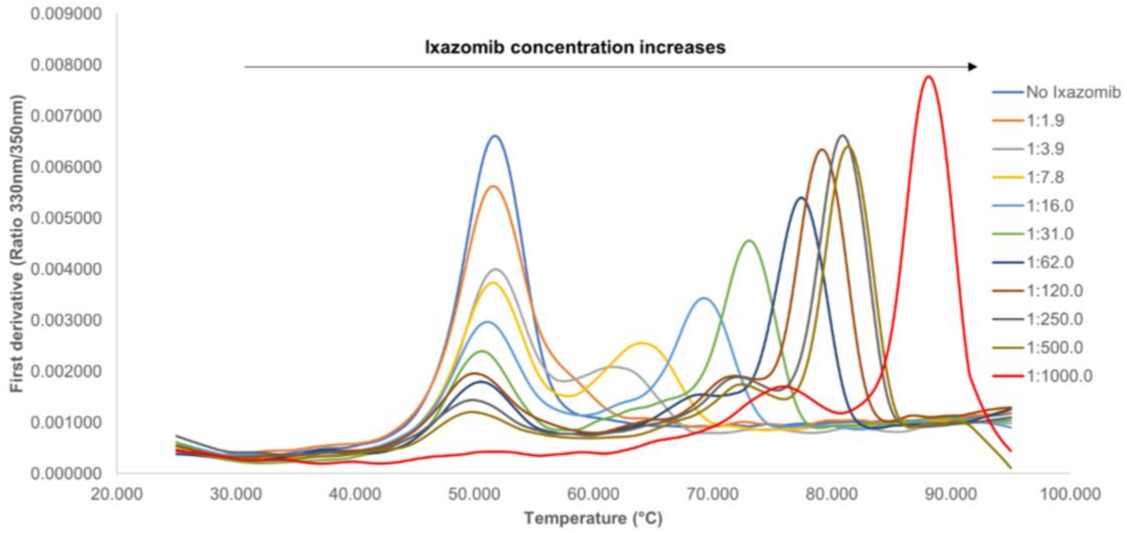

b

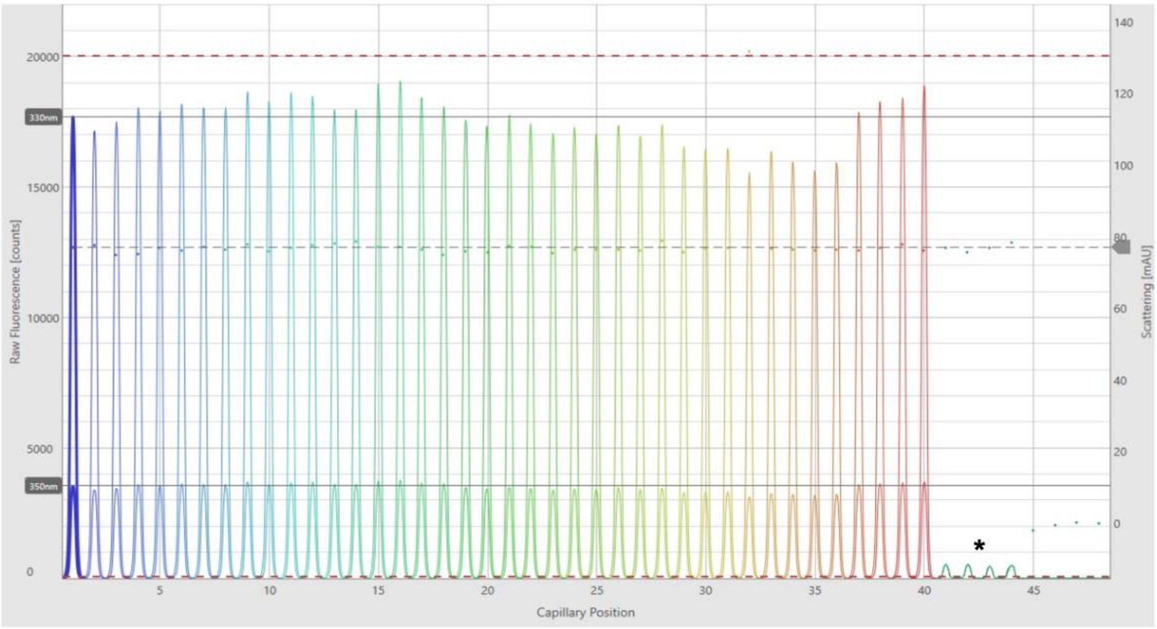

Fig. S4 – (**a**) nanoDSF measurements with different concentrations of ixazomib and a ligand-free control with 1% v/v DMSO ("No Ixazomib" curve). This final DMSO concentration was the same in all the samples. (**b**) The "Discovery Scan" graph was built by the PR.ThermControl Software (NanoTemper, Germany) is used to detect the fluorescence intensity in each capillary. In the last four positions (marked with an *), the samples only contain ixazomib and buffer, and based on the values of raw fluorescence of the four peaks (1000 μM ixazomib), it is demonstrated that the ligand did not affect the measurements.

Table 2 – Experimental data of SAXS measurements.

	**Heptameric** *Se***ClpP** (P2)	**Tetradecameric** *Se***ClpP** (P1)	*Se***ClpP – ixazomib**
**Data collection parameters**			
X-ray source	PETRA III, EMBL P12 beamline		
Wavelength (nm)	0.124		
Detector distance (m)	3.1		
Temperature (K)	293		
**Structural parameters**			
I(0) (*P(r)*function)	553.00 ± 0.24	1011.00	1840.00 ± 0.93
I(0) (Guinier/AutoR$_G$)	553.00 ± 0.76	1018.21±0.91	1840.00 ± 5.35
I(0) (SAXSMoW [1])	412.40	1015.57	1842.88
R$_G$ (nm) (*P(r)* function)	3.78 ± 0.008	4.38 ± 0.002	4.50 ± 0.02
R$_G$ (nm) (Guinier/AutoR$_G$)	3.75 ± 0.003	4.47±0.006	4.59 ± 0.002
R$_G$ (nm) (SAXSMoW [1])	3.73	4.46	4.57
qR$_G$ limit (Guinier/AutoR$_G$)	1.34	1.30	1.33
qR$_G$ limit (SAXSMoW [1])	1.30	1.29	1.30
D$_{max}$ (nm) (*P(r)* function)	11.00	12.43	12.60
Porod volume estimate (Å^3)	186890	395281	476010
P = R$_G$/R$_H$ [2]	0.75	0.80	0.76
χ^2 value, CorMap P-value	1.07, 0.93	1.00, 0.92	1.07, 0.92
Molecular mass (kDa) (SAXSMoW [1])	135.00	288.53	318.00
Molecular mass (sequence)	149.57	299.15	299.15
Oligomeric state	Heptamer	Tetradecamer	Tetradecamer

[1] SAXSMoW is an online server provided for analysis of SAXS data offered by the University of São Paulo, Instituto of Physics, São Carlos, Brazil.

[2] R$_H$ is hydrodynamic radius measured by Dynamic Light Scattering (DLS).

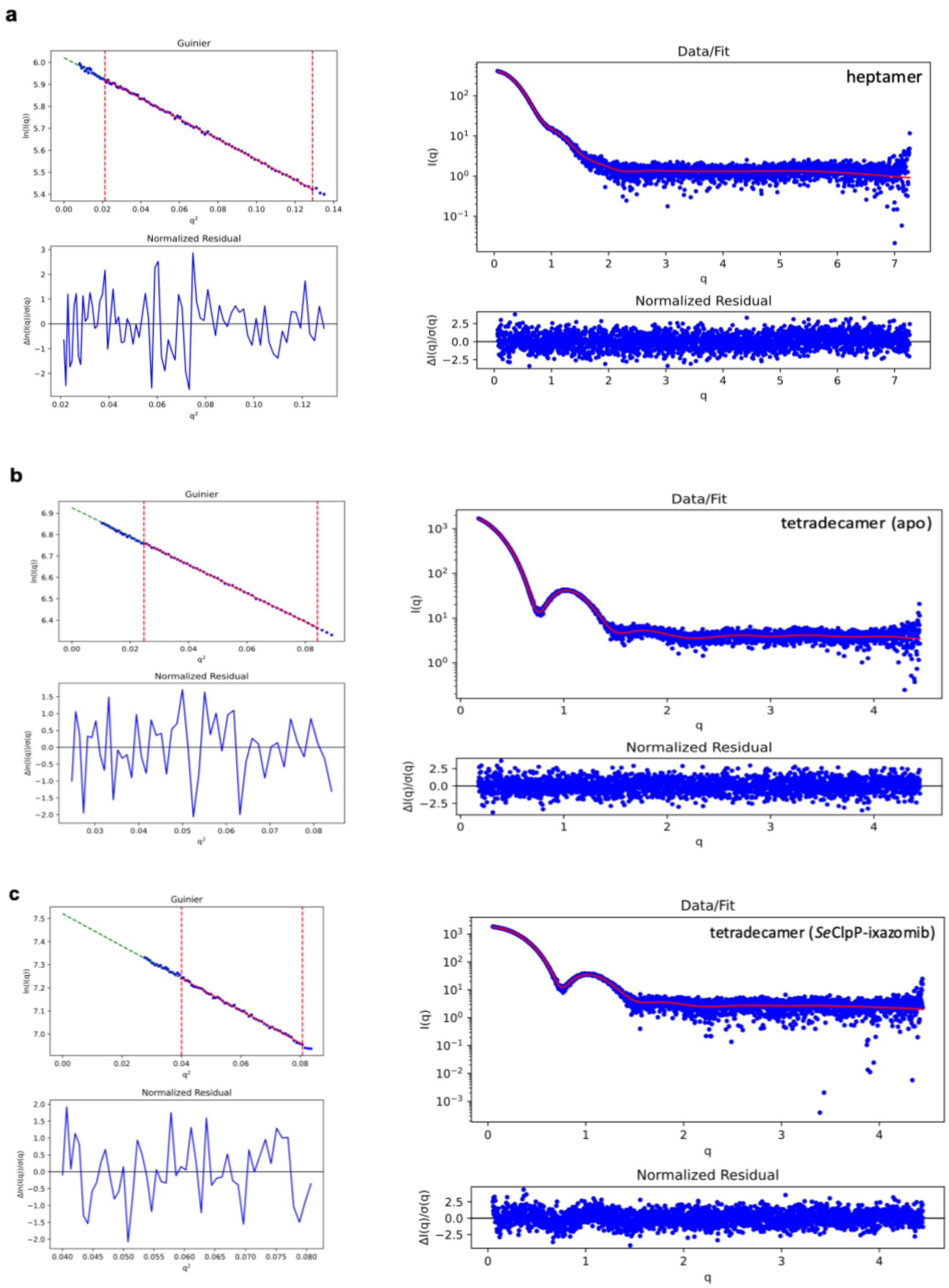

Fig. S5 – Guinier plot and scattering intensity graph (I(q) *vs* q) of SAXS measurements after size-exclusion chromatography (SEC): (**a**) heptameric apo *Se*ClpP, (**b**) tetradecameric apo *Se*ClpP and (**c**) tetradecameric *Se*ClpP-ixazomib complex. The graphs were built on BioXTAS RAW software[38].

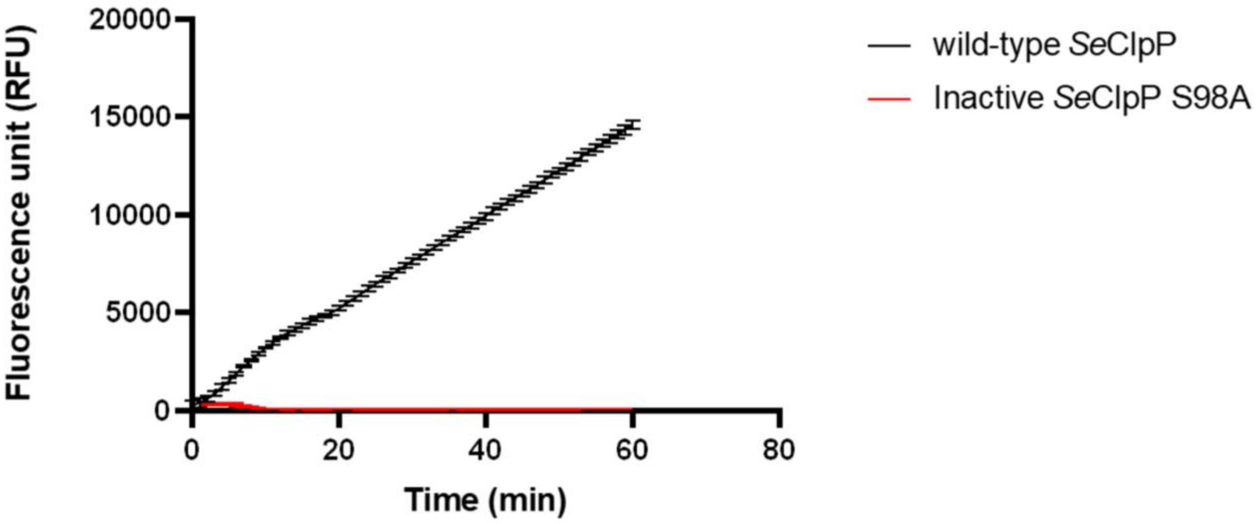

Fig. S6 - Hydrolysis of the fluorogenic peptide substrate Suc-LY-AMC (100µM) by wild-type *Se*ClpP (1µM) and the inactive mutant *Se*ClpP S98A (1µM). In the mutant, the active Ser98 was replaced by alanine (Ala98). The red curve indicates that there is no presence of endogenous *Ec*ClpP in the purified protein sample, as no residual peptidolytic activity was observed. The black curve shows the substrate degradation caused by the wild-type *Se*ClpP. Both wild-type *Se*ClpP and *Se*ClpP S98A were expressed and purified by applying the same procedures. The cleavage of Suc-LY-AMC was monitored by fluorescence (excitation = 340 nm; emission = 460 nm). Error bars represent the standard deviation of three independent measurements.
